# Character displacement or priority effects: immigration timing can affect community assembly with rapid evolution

**DOI:** 10.1098/rspb.2024.2145

**Published:** 2024-11-20

**Authors:** Keiichi Morita, Masato Yamamichi

**Affiliations:** ^1^ Department of General Systems Studies, The University of Tokyo, 3-8-1 Komaba, Meguro, Tokyo 153-8902, Japan; ^2^ School of Biological Sciences, The University of Queensland, St Lucia, Brisbane, Queensland 4072, Australia; ^3^ Department of International Health and Medical Anthropology, Institute of Tropical Medicine, Nagasaki University, Nagasaki 852-8523, Japan

**Keywords:** community assembly, community monopolization, ecological character displacement, evolution-mediated priority effect, rapid evolution, resource competition

## Abstract

Understanding how biological communities assemble in the presence of rapid evolution is becoming an important topic in ecology. Previous studies demonstrated that community assembly can be affected by two types of eco-evolutionary dynamics: evolution-mediated priority effect (EPE) and ecological character displacement (ECD). In EPE, early-arriving species prevent colonization of late-arriving species via local adaptation (i.e. community monopolization), whereas ECD promotes species coexistence by niche partitioning. Researchers tended to discuss the two processes separately, but it should be possible for those processes to operate in the same system depending on various conditions. Here, we developed a theoretical framework that integrates the two processes by using a simple two-species competition model with eco-evolutionary feedback. We revealed that, when an early-arriving species evolves, the difference in immigration timing between the early-arriving and a late-arriving species can be a key parameter. When the difference is small, ECD occurs because insufficient local adaptation of the early-arriving species allows colonization of the late-arriving species. When the difference is large, however, EPE occurs because niche pre-emption by local adaptation of the early-arriving species prevents colonization of the late-arriving species. Further theoretical and empirical studies will be important to better understand eco-evolutionary community assembly with ECD and EPE.

## 1. Introduction

**Figure 1.**
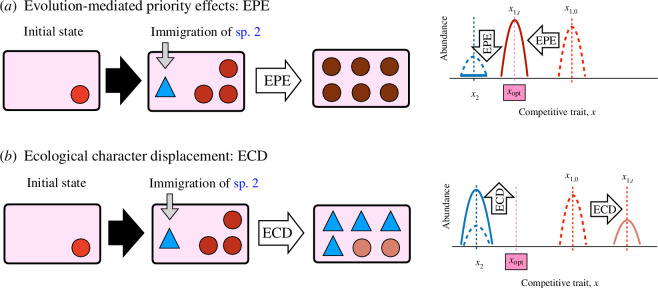
Concepts of evolution-mediated priority effect (EPE) and ecological character displacement (ECD). (*a*) EPE occurs when rapid adaptation of an early-arriving species 1 to a local environment can prevent colonization of a late-arriving species 2. The circles and triangles represent species 1 and 2, respectively, and the background rectangle is a local habitat. Dark colour indicates locally adapted states. Adaptation to the local habitat of species 1 moves the species 1’s trait *x*_1_ from its initial state, shown by the dashed red curve, to *x*_opt_, as shown by the solid thick red curve. Local adaptation of species 1 causes competitive exclusion of species 2 (i.e. the abundance of species 2 goes to zero). (*b*) ECD occurs when the divergence of competitive traits weakens interspecific resource competition and promotes stable coexistence. ECD moves the species 1’s trait *x*_1_ away from the optimal trait value, *x*_opt_, and it promotes coexistence.

One of the major challenges in ecology is to understand factors determining species composition in a local habitat through community assembly [1–6]. Previous studies have revealed that various ecological and evolutionary factors can affect community assembly, including environmental filtering [7], interspecific competition [8], immigration history [9], adaptive radiation [10] and demographic stochasticity [11]. Recently, increasing evidence indicates that rapid contemporary evolution plays an important role in community assembly [4,6,12] mainly via two processes: (i) evolution-mediated priority effects (EPE), where early-arriving species rapidly adapt to local environments and monopolize a niche (community monopolization: figure 1*a*) [5,13–18], and (ii) ecological character displacement (ECD), where trait divergence results in niche partitioning between competing species and promotes stable coexistence (figure 1*b*) [19–27].

Spatial heterogeneity in environments and local adaptation are pervasive in the wild [28], and rapid evolution to novel environments in local habitats can affect community assembly through EPE: rapid adaptation of an early-arriving species to a local environment can prevent colonization of a late-arriving species [5]. Previous theoretical studies suggested that EPE can be an important factor in determining community assembly under various circumstances [13–16]. A recent experimental study demonstrated that adaptation of an early-arriving species reduces the abundance of a late-arriving species by 63% relative to the cases of simultaneous arrival and no adaptation by using two archaeal species in different temperatures [18]. On the other hand, ECD occurs via trait divergence that weakens interspecific resource competition and promotes stable coexistence [19,21–23,26]. Although classical studies have assumed that ECD is a consequence of long-term evolution [21,26,29], accumulating empirical studies have demonstrated that ECD can occur rapidly enough to affect contemporary population dynamics [19,21,22,24,26,27,30]. A recent experimental study on the bacterium *Pseudomonas fluorescens* has shown that short-term sympatric evolution can weaken priority effects, possibly owing to niche differentiation [24].

Previous studies tended to consider how rapid evolution can affect community assembly by focusing on either EPE [13–16,18] or ECD [24,27], but to our knowledge, no study has synthesized the two processes. It is important to synthesize them because EPE and ECD are very different outcomes generated by the same general processes, and we do not understand when we should see EPE and ECD. Here, we propose a theoretical framework integrating EPE and ECD and demonstrate that immigration timing can be a key parameter for the occurrence of EPE or ECD. By analysing three models for two-species competition, we show that a small difference in immigration timing can cause ECD, whereas a large difference in immigration timing can result in EPE as it allows the early-arriving species to adapt to local environments.

## 2. Models

We consider a discrete-time model of two competing species with population densities *N*_1,*t*_ and *N*_2,*t*_ [31] in the main text:

(2.1) $$N_{i,t+1}=\frac{\lambda_{i}\left(x_{i,t}\right)N_{i,t}}{1+\alpha_{ii}N_{i,t}+\alpha_{ij}\left(x_{i,t},x_{j,t}\right)N_{j,t}},\;\;\;\left(i,j=1,2\right),$$

where *λ_i_* is the *per capita* fecundity, and *α_ii_* and *α_ij_* are the strengths of intraspecific and interspecific competition, respectively. This Leslie–Gower model was used to describe competition dynamics in annual plants and insects [27,31–35]. Its dynamics serve as a discrete-time analogue of the classical continuous-time Lotka–Volterra competition model [36]. We consider frequency-dependent dynamics of the Leslie–Gower model in electronic supplementary material, appendix S1, and density-dependent dynamics of the continuous-time Lotka–Volterra and consumer-resource models in electronic supplementary material, appendices S2 and S3.

We assume species *i* has a quantitative trait with the average value, *x_i_* (*i* = 1, 2), and the difference between the mean trait values of the two species determines the strength of interspecific resource competition as follows:

(2.2) $$\alpha_{ij}\left(x_{i,t},x_{j,t}\right)=\alpha_{ij,max}exp\left[-{\left(x_{i,t}-x_{j,t}\right)}^{2}\right],\;\;\left(i,j=1,2\right),$$

where *α_ij_*_,max_ is the maximum strength of interspecific resource competition. In addition, the *per capita* fecundity is assumed to be maximized at a trait value $x_{\lambda }$ as follows:

(2.3) $$\lambda_{i}\left(x_{i,t}\right)=\lambda_{i,max}exp\left[-s_{i}{\left(x_{i,t}-x_{\lambda }\right)}^{2}\right],\;\;\;\left(i=1,2\right),$$

where *λ*_*i*,max_ is the maximum *per capita* fecundity and *s*_*i*_ is the scaling parameter for trait divergence (or the strength of stabilizing selection, i.e. increasing *s*_*i*_ makes *λ*_*i*_ more sensitive to trait divergence). Thus, without interspecific competition, adaptive evolution will maximize the *per capita* fecundity by moving the trait mean *x*_*i*_ to $x_{\lambda }$. However, the presence of a competing species can introduce a trade-off between weakening interspecific competition (i.e. increasing the trait difference |*x*_*i*_ *− x*_*j*_|) and increasing the *per capita* fecundity (i.e. decreasing the difference |*x*_*i*_ − $x_{\lambda }$|, electronic supplementary material, figure S1). Increasing the trait difference between the two species weakens interspecific competition (character displacement: [27,37–39]), but it may simultaneously decrease the *per capita* fecundity. There may be an equilibrium trait value where adaptive evolution stops owing to a balance between weakening interspecific competition and decreasing the *per capita* fecundity.

Assuming that many autosomal loci affect the quantitative traits additively and that the trait distribution in a population concentrates sharply around the population average, adaptive evolution of the quantitative trait mean occurs along the fitness gradient as follows [40]:

(2.4) $$x_{i,t+1}=x_{i,t}+G_{x,i}\frac{\partial lnW_{i}}{\partial x_{i,t}},\;\;\;\left(i=1,2\right),$$

where *W_i_* = *N_i_*_,*t*+1_/*N_i_*_,*t*_ is the fitness of species *i*, and *G_x_*_,*i*_ is additive genetic variance of a competitive trait of species *i* (*i* = 1, 2). The direction of evolution is determined by the fitness gradient, while the speed of evolution is determined by the fitness gradient and additive genetic variance. Although the original study assumed weak selection for deriving equation (2.4) [40], recent theoretical studies have shown that the model is useful to describe rapid evolution and to understand eco-evolutionary dynamics where selection is strong [27,41–47]. Because we consider a situation where species 1 evolves and species 2 does not hereafter, we represent species 1’s additive genetic variance *G_x_* instead of *G_x_*_,1_. We regard species 1 as a resident species when species 1 is abundant, but as an immigrant species when species 1 is rare.

Following previous theoretical studies on eco-evolutionary dynamics (e.g. [27,41–49]), we assume constant additive genetic variance. We assume that the trait value that maximizes the *per capita* fecundity, $x_{\lambda }$, is 0 and that species 2 has a constant trait mean at *x*_2_ = −0.1 owing to, for example, continuous immigration from other habitats or depleted genetic variance due to immigration. The initial trait value of species 1 is assumed to be positive and maladaptive (*x*_1,0_ = 0.5). The initial density of species 1 is assumed to be at its carrying capacity (i.e. *N*_1,0_ = (*λ*_1_ – 1)/α_11_) and that of species 2 is assumed to be zero until immigration and to be small at the arrival timing (*N*_2*,t*_ = *N*_1,0_/10). When the maximum *per capita* fecundity λ_*i*,max_ is large enough, density-dependent competition dynamics can be approximated to frequency-dependent dynamics [35] to be better visualized for understanding stable coexistence (i.e. negative frequency dependence) [33,50]. Through this approximation, we reduce the number of variables in the model from three (i.e. *N*_1*,t*_*, N*_2*,t*_ and *x*_1*,t*_) to two by considering species 1’s frequency, *M*_*t*_ = *N*_1*,t*_/(*N*_1*,t*_+ *N*_2,*t*_) (electronic supplementary material, appendix S1a) for drawing nullclines where dynamics equilibrate assuming that the *per capita* fecundity is large [35]. We used Mathematica [51] for analysing models and R for numerical iterations of the recursion equations [52].

## 3. Results

**Figure 2.**
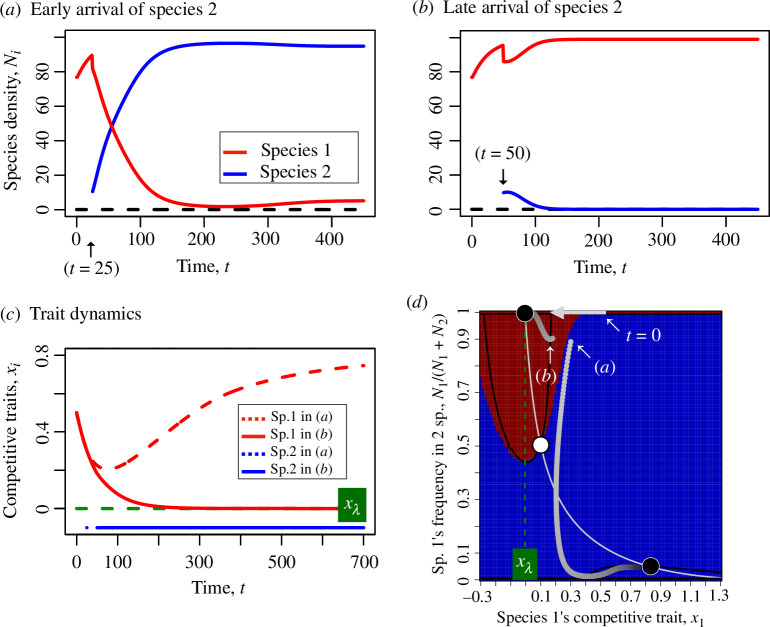
Population and trait dynamics of two species and a nullcline plot. (*a*) When the difference in immigration timing is small, ecological character displacement (ECD) occurs and an early-arriving species 1 stably coexists with a late-arriving species 2. The population density of species 1 is low owing to maladaptation to a local habitat. (*b*) When the difference is large, the early-arriving species 1 is locally adapted and prevents establishment of the late-arriving species 2 by driving extinction of species 2 (i.e. evolution-mediated priority effect, EPE). (*c*) When the difference in immigration timing is small, colonization of species 2 prevents a competitive trait of species 1, *x*_1_, to approach the optimum trait maximizing the *per capita* fecundity, *x*_*λ*_, and causes *x*_1_ to diverge from species 2’s trait, *x*_2_, to avoid interspecific competition (i.e. ECD) (red dashed line). On the other hand, when the difference is large, *x*_1_ approaches *x*_*λ*_, which promotes extinction of species 2 (i.e. EPE) (red solid line). (*d*) The nullclines show alternative stable states including two locally stable equilibria (black points): one where species 2 goes extinct when *x*_1_ reaches *x*_*λ*_ and the other where species 1 and 2 stably coexist owing to trait divergence. A white circle represents a locally unstable equilibrium. Black and grey lines are nullclines of species 1’s frequency and trait evolution, respectively. Grey points are results of numerical iterations of the recursion equations shown in (*a*), (*b*), and (*c*), and the arrow ‘*t* = 0’ indicates the initial condition. The red and blue regions indicate the basins of attraction towards the extinction and coexistence equilibria, respectively. Parameter values are $\alpha_{ii}=s_{i}=1$, $\alpha_{12,max}=\alpha_{21,max}=1.1$, $x_{2}=-0.1$, $x_{\lambda }=0$, $\lambda_{i,max}=100$ (*i* = 1, 2) and *G*_*x*_ = 0.01, and initial conditions are $x_{1,0}=0.5$, $N_{1,0}=(\lambda_{1}-1)/\alpha_{11}$ and *N*_2,0_ = 0 until the immigration timing of species 2, *t*_*i*_, and $N_{2,t_{i}}=N_{1,0}/10$ where *t*_*i*_ = 25 or 50.

We found that the immigration timing of the late-arriving species (species 2) can change eco-evolutionary dynamics (figure 2; electronic supplementary material, figure S2). When the immigration timing of the non-evolving species 2 is early (*t* = 25), the two species stably coexist (figure 2*a*) because of trait divergence (ECD: figure 2*c*; electronic supplementary material, figure S3a,b). On the other hand, when the immigration timing of the non-evolving species 2 is late (*t* = 50), species 1 dominates and species 2 goes extinct (figure 2*b*) because species 1 maximizes its *per capita* fecundity (EPE: figure 2*c*).

**Figure 3.**
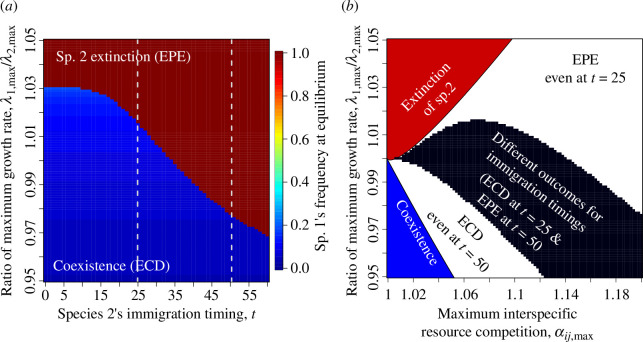
Effects of immigration timing on coexistence. (*a*) When the immigration timing of species 2 is early and the maximum growth rate (*per capita* fecundity) of species 2 is large, coexistence occurs (i.e. ecological character displacement, ECD). On the other hand, when the immigration timing of species 2 is late and the maximum growth rate of species 2 is small, extinction of species 2 is more likely (i.e. evolution-mediated priority effect, EPE). Two vertical dashed lines show the immigration timings (*t* = 25 and 50) in figures 2 and 3*b*. (*b*) Alternative stable states emerge when the maximum strength of interspecific resource competition (*α*_*ij*,max_) is large and the maximum *per capita* fecundities of species 1 (*λ*_1,max_) and species 2 (*λ*_2,max_) are almost equivalent (white and black regions). The black region indicates parameter conditions where immigration of species 2 at *t* = 25 results in coexistence (ECD as figure 2*a*), whereas immigration of species 2 at *t* = 50 results in extinction of species 2 (EPE as figure 2*b*). The white region above the black region shows extinction of species 2 even when species 2 arrives at *t* = 25. The white region below the black region shows coexistence even when species 2 arrives at *t* = 50. See electronic supplementary material, figure S7, for the shape of the black region with different immigration timings. When *α*_*ij*,max_ is small and the fecundity ratio is large (the red region), extinction of species 2 occurs irrespective of the initial condition (i.e. the stable equilibrium of coexistence disappears, but the stable equilibrium without species 2 remains). When *α*_*ij*,max_ and the fecundity ratio are both small (the blue region), coexistence occurs irrespective of the initial condition (i.e. the stable equilibrium without species 2 disappears, and there is the stable equilibrium of coexistence). The other parameters are the same as in figure 2.

The eco-evolutionary dynamics with alternative stable states can be illustrated by a nullclines analysis along the trait value of species 1 and the frequency of species 1 (figure 2*d*; electronic supplementary material, appendix S1). Here, the black and grey curves represent conditions where species 1’s frequency and species 1’s trait equilibrate, and their intersections are equilibria. There are two locally stable equilibria (black points): one where species 2 goes extinct and the trait evolves to the optimal value (*M*, *x*_1_) = (1, *x_λ_*), and the other where the two species coexist and the trait diverges from *x_λ_*, (*M*, *x*_1_) = ($\overset{-}{M},{\overset{-}{x}}_{1}$) (figure 2*d*). When we assume the initial condition is at the top (*t* = 0) and the difference in immigration timing is small (figure 2*a*), the eco-evolutionary dynamic leaves a basin of attraction towards the extinction equilibrium of species 2 (the red region) and moves to the coexistence equilibrium (the bottom-right point). On the other hand, when the difference in immigration timing is large (figure 2*b*), the eco-evolutionary dynamic enters the basin of attraction and eventually moves to the extinction equilibrium of species 2 (the top-left point). We arbitrarily chose *t* = 25 and 50 for the arrival timing of species 2 in figure 2, but we considered a broader range of immigration timings in figure 3*a* (see also electronic supplementary material, figure S4).

The above alternative stable states emerge when the maximum strength of interspecific resource competition, *α_ij_*_,max_, is large and the maximum *per capita* fecundities of two species (*λ*_1,max_ and *λ*_2,max_) are equivalent (the white and black regions in figure 3*b*). When *α_ij_*_,max_ is small and *λ*_1,max_ is larger than *λ*_2,max_, the coexistence equilibrium becomes unstable (the top-left red region in figure 3*b*), whereas the extinction equilibrium of species 2 becomes unstable when *α_ij_*_,max_ is small and *λ*_1,max_ is smaller than *λ*_2,max_ (the bottom-left blue region in figure 3*b*; electronic supplementary material, figure S5 and appendix S1b). The different outcomes due to species 2’s arrival at *t* = 25 and 50 (figure 2) arise in the black region in figure 3*b*. In the white region above the black region, EPE occurs even when species 2 arrives at *t* = 25, whereas in the white region below the black region, ECD occurs even when species 2 arrives at *t* = 50 (figure 3*b*). Changing the immigration timings from *t* = 25 and 50 alters the shape of the black region (electronic supplementary material, figures S6 and S7). Changing parameter values of *α_ij_*_,max_ and *λ*_1,max_/*λ*_2,max_ alters the shapes of nullclines (electronic supplementary material, figure S8) and the basins of attraction (electronic supplementary material, figure S9). Increasing genetic variance *G_x_* broadens the basin of attraction to the extinction equilibrium of species 2 owing to faster evolution of species 1 (electronic supplementary material, figure S10).

Results in the discrete-time Leslie–Gower model shown in the main text are qualitatively similar to those in the continuous-time Lotka–Volterra competition model (electronic supplementary material, figures S2 and S3c,d) and in the consumer-resource model. Readers may wonder how alternative stable states (i.e. interspecific competition is stronger than intraspecific competition) arise in the consumer-resource model. Thus, we first clarified the condition for ecological priority effects in the consumer-resource model (i.e. when the conversion efficiencies of consumers’ main resources are small: electronic supplementary material, figure S11) and then demonstrated that the model showed quantitatively similar results to the Leslie–Gower and Lotka–Volterra models (electronic supplementary material, figures S12 and S13). In addition, we visualized how our results can be interpreted through the lens of coexistence theory (electronic supplementary material, figure S14).

We also checked the robustness of our analyses in terms of (i) the initial population densities (electronic supplementary material, figure S15), (ii) the approximated frequency dynamics (electronic supplementary material, figure S16), and (iii) the strength of selection and genetic architecture (electronic supplementary material, figure S17). First, we found that the population density of species 1 quickly increases towards its carrying capacity even when the maximum fecundity is relatively small (*λ*_*i*,max_ = 10 in electronic supplementary material, figure S15). Therefore, the assumption that the initial density of species 1 is its carrying capacity is not critical. We also showed that ECD is more likely when the density of species 2 at the arrival timing is large even when the arrival timing is late (electronic supplementary material, figure S15e). Second, we showed that the original density dynamics cannot be well approximated to the frequency dynamics when the maximum fecundity is small as the coexistence equilibrium is lost in the original model (electronic supplementary material, figure S16). Thus, it should be noted that the analyses based on the approximated frequency dynamics (figure 2*d*) are meaningful only when the assumption of large fecundity is satisfied. Third, we considered a clonal model where species 1 has two clonal genotypes with trait values 0 (locally adapted but experiences strong interspecific competition) and 0.5 (locally maladapted but experiences weak interspecific competition). The clonal model has also been used to understand eco-evolutionary dynamics (e.g. [41]) and does not require the weak-selection assumption. When the maladaptive clone is initially common, we found that the clonal model can show similar eco-evolutionary community assembly dynamics to the quantitative genetic model (electronic supplementary material, figure S17). This suggests that genetic architecture may not be crucial for our conclusion, and the fitness gradient model based on quantitative genetics [40] is useful for understanding rapid evolution where selection is strong [27,41–47].

## 4. Discussion

### (a) Synthesizing evolution-mediated priority effect and ecological character displacement

We proposed a theoretical framework for understanding how rapid evolution can affect community assembly by synthesizing two processes: evolution-mediated priority effect (EPE) and ecological character displacement (ECD). We demonstrated that the immigration timing of a late-arriving species can alter the direction of rapid evolution in an early-arriving species and the eco-evolutionary outcomes (i.e. ECD and stable coexistence or EPE and extinction of non-evolving species 2). While previous studies on EPE have considered the possible importance of immigration timing [14], they did not consider ECD. Similarly, previous studies on ECD tended not to consider a situation where interspecific competition is stronger than intraspecific competition, which causes priority effects [53]. Our study suggests that the difference in immigration timing has a crucial effect on the occurrence of EPE and ECD under a trade-off between interspecific competition and the *per capita* fecundity. Although readers may think that it is rare to see a situation where the second species arrives during the process of local adaptation of the first species, recent studies have shown that evolutionary processes (e.g. local adaptation) and ecological processes (e.g. immigration) occur on the same timescale, and thus this is a meaningful setup to understand eco-evolutionary dynamics and clarify when EPE or ECD will occur. It will be interesting to conduct laboratory experiments with bacteria [9,24], archaea [18], plankton [5] and other organisms to test our theoretical predictions.

The trade-off between weakening interspecific interactions and increasing fecundity was reported in empirical studies of character displacement. For example, in spadefoot toads, declining body sizes caused niche partitioning and decreased the number of eggs and survival rate [54]. Furthermore, previous studies on Darwin’s finches suggest a similar trade-off [20]. Further empirical research will be important for understanding the shape of the trade-off. In addition to the trade-off between interspecific competition and fecundity in our model, we can consider a different form of trade-off between intra- and interspecific competition. Empirical studies demonstrated that this trade-off between intra- and interspecific competitive ability is plausible in allelopathy of plants [55], and theoretical studies highlighted that rapid evolution along the trade-off can promote stable coexistence with population cycles [43,49,56–58]. Although cyclic dynamics is out of scope in this study, it will be significant to investigate more complex eco-evolutionary dynamics.

We considered character displacement with alternative stable states in this study. Previous studies have revealed that alternative stable states can strongly affect ecological dynamics via, for example, priority effects and hysteresis [4,53,59]. Many studies have recognized the potential importance of ecological priority effects arising from positive frequency dependence where there are two locally stable equilibria [4,53]. However, studies on character displacement did not consider the situation where interspecific resource competition is stronger than intraspecific resource competition [27,37,38]. With nonlinear interactions, various alternative stable states arise and immigration timing can become important [60], as our model suggests.

Recent studies have pointed out that Chesson’s modern coexistence theory is an important theoretical framework for understanding species competition [8,23,39,61,62]. In the framework of modern coexistence theory, species coexistence can be understood through the balance between niche and competitive ability differences [61]. Although most previous studies have adopted the framework for ecological dynamics, recent studies have proposed that it can integrate rapid evolution as well [39,58]. When we employ the framework, the initial conditions are placed in a region of ecological priority effects (electronic supplementary material, figure S14), and then the system either moves to a stable coexistence region through ECD or stays in the priority effects region (i.e. EPE). It should be noted that the niche and competitive ability differences are not independent, as a recent study pointed out [63], because ECD and EPE simultaneously change them along the assumed trade-off.

It will be possible to introduce some additional processes into our model to consider more realistic dynamics in nature. First, our models assume that additive genetic variance is constant. Previous theoretical studies suggest that additive genetic variance can change dynamically during ECD [37,38,64]. Thus, it will be important to conduct simulations with genetically explicit processes [64] and individual-based models in addition to the clonal model (electronic supplementary material, figure S17) to examine how additive genetic variance changes can affect coexistence. Second, it will be possible to introduce demographic and environmental stochasticity. Demographic stochasticity is important to investigate how extinction occurs with ECD and EPE. Furthermore, recent studies suggest that changing environments can affect species distribution as well as community structure and stability [17]. Thus, it will be essential to consider environmental fluctuation to examine how global change will affect community assembly when investigating extinction of native species. Third, we considered a situation where only an early-arriving species evolves, but coevolution is more likely in nature. Coevolution of two species may produce complex eco-evolutionary dynamics [42,43,45] and detailed analyses are needed to examine how coevolution can affect coexistence. Finally, it will be important to consider community dynamics with three or more species. Dynamics with three or more species may produce more complex dynamics including alternative stable states [60,65] and chaotic population cycles [66,67]. Although we considered two-species dynamics with a single niche axis as a starting point, it will be fruitful to construct models with multiple species and multiple niche axes to generalize our results.

### (b) Conclusion

In conclusion, we showed that the difference in immigration timing between two competing species can change the direction of rapid evolution, resulting in different ecological outcomes (i.e. either coexistence or extinction of late-arriving species). Our simple modelling framework may become a basis for understanding how rapid evolution can affect community assembly by integrating EPE and ECD. Owing to rapidly changing environments and species distribution, it will be important to develop an integrative framework for understanding complex eco-evolutionary dynamics and coexistence of early- and late-arriving species for conservation and wildlife management. We expect that our models will be an important step towards a comprehensive understanding of how rapid evolution can affect community dynamics.

## Data Availability

Our code is uploaded as electronic supplementary material [68].
